# Intravitreal Aflibercept as a Rescue Therapy for Retinal Neovascularization and Macular Edema due to Eales Disease

**DOI:** 10.1155/2021/8887362

**Published:** 2021-02-11

**Authors:** Ning-Yi Hsia, Chun-Ju Lin, Chun-Ting Lai, Henry Bair, Cheng-Hsien Chang, Jane-Ming Lin, Yi-Yu Tsai

**Affiliations:** ^1^Department of Ophthalmology, China Medical University Hospital, China Medical University, Taichung, Taiwan; ^2^School of Medicine, College of Medicine, China Medical University, Taichung, Taiwan; ^3^Department of Optometry, Asia University, Taichung, Taiwan; ^4^Stanford University School of Medicine, Stanford, CA, USA

## Abstract

We report the rescue effect of intravitreal aflibercept injections on retinal neovascularization and macular edema due to Eales disease. Case 1 was a 36-year-old female. Intravitreal aflibercept was administered as rescue therapy after persistent retinal neovascularization following retinal photocoagulation, periocular triamcinolone, and intravitreal ranibizumab injection. Retinal neovascularization initially regressed, but recurred after 5 months along with macular edema. Two more intravitreal aflibercept injections were given, and retinal neovascularization with macular edema regressed. Her vision improved to 20/25 and remained stable after 43 months. Case 2 was a 27-year-old female. Intravitreal aflibercept was administered after persistent retinal neovascularization and macular edema following periocular triamcinolone injection. The macular edema initially subsided but recurred after 3 months. Intravitreal aflibercept injections were then administered once every three months to maintain her vision 20/20. The patient has been followed up for 28 months. Intravitreal aflibercept was effective as a rescue therapy in the treatment of Eales disease to regress retinal neovascularization, though repeated injections were necessary in cases of recurrence.

## 1. Introduction

Eales disease is a potentially blinding idiopathic retinal vasculopathy, involving inflammatory venous occlusion that primarily affects the peripheral retina of healthy young adults. Retinal changes include periphlebitis, peripheral nonperfusion, and neovascularization. In addition, visual loss is characteristically caused by recurrent vitreous hemorrhage [1–3].

The presence of inflammatory cytokines (interleukin-6, interleukin-8, and monocyte chemoattractant protein-1) and vascular endothelial growth factor (VEGF) in the vitreous has previously been observed in Eales disease [4, 5]. Angiogenesis is a highly complex and coordinated process in which VEGF plays a pivotal role, under both normal and pathological conditions. Since VEGF is upregulated during retinal hypoxia, anti-VEGF agents are now being used for treatment of pathological ocular angiogenesis and macular edema.

We report the rescue effects of intravitreal aflibercept (EYLEA, Regeneron Pharmaceuticals, Tarrytown, NY) injections in two cases of retinal neovascularization and macular edema in Eales disease. These two cases had persistent retinal neovascularization with macular edema after focal retinal photocoagulation, periocular depot triamcinolone, or intravitreal ranibizumab treatment. To our knowledge, this is the first report describing the use of intravitreal aflibercept in a relatively advanced stage of Eales disease.

## 2. Case Presentation

We retrospectively reviewed two patients with retinal neovascularization (NV) and macular edema (ME) due to Eales disease, who were treated with intravitreal 2.0 mg/0.05 mL aflibercept injections as rescue therapy. Diagnosis and assessment of Eales disease were based on the findings of fluorescein angiography (FA) and negative interferon-gamma release assays (QuantiFERON®-TB test). Magnetic resonance imaging (MRI) was also performed to differentiate optic neuritis-induced retinal NV. They had no significant past medical history either physical signs of mucosal ulceration or arthritis. Their laboratory study for ruling out infection and autoimmune disease, such as complete blood counts, HLA-B27, HLA-B51, antiphospholipid antibodies, antinuclear autoantibodies, antineutrophil cytoplasmic autoantibodies, anti-dsDNA, anti-Sm, or rheumatoid factor, was all negative results.

**Figure 1:**
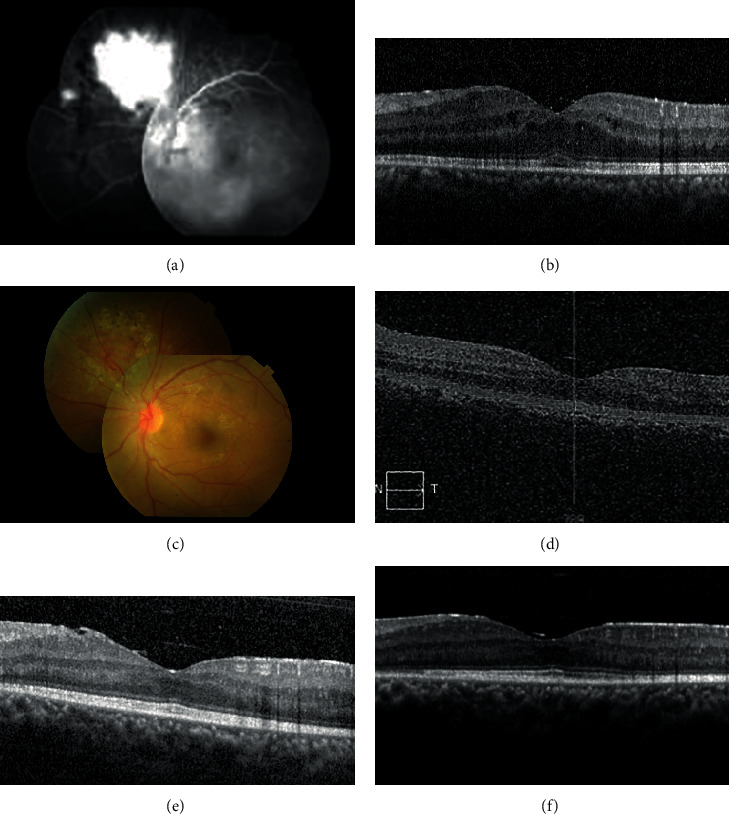
(a) FA showed vascular tortuosity, peripheral retinal neovascularization (NV), and macular edema (ME). (b) OCT revealed ME (central foveal thickness 376 *μ*m). (c) After the first intravitreal aflibercept injection, the retinal NV regressed. (d) ME also decreased (central foveal thickness 293 *μ*m). (e) ME recurred 5 months after the first intravitreal aflibercept injection (central foveal thickness 305 *μ*m). (f) No recurrent ME 15 months after the third intravitreal aflibercept injection (central foveal thickness 295 *μ*m).

Case 1 was a 36-year-old female with presenting BCVA counting fingers OS due to vitreous hemorrhage (VH). Oral transamin and topical steroid treatment were given. FA showed vascular tortuosity, peripheral retinal NV, and ME (Figure 1(a)). OCT also revealed ME (Figure 1(b)). Intravitreal aflibercept was used as rescue therapy due to persistent retinal NV and ME after administration of focal retinal photocoagulation, periocular depot triamcinolone acetonide, and intravitreal ranibizumab injections. After the first intravitreal aflibercept injection, the retinal NV regressed (Figure 1(c)) and ME decreased (Figure 1(d)). ME recurred after 5 months (Figure 1(e)). Supplemental retinal photocoagulation was performed, and the second intravitreal aflibercept injection was given. The retinal NV regressed again and ME resolved. Recurrent VH was noted after 15 months, and a third intravitreal aflibercept injection was given. VH and ME were then resolved (Figure 1(f)). The patient's BCVA improved to 20/25 and was followed up for 43 months. Her intraocular pressure was within normal limits during the whole course.

**Figure 2:**
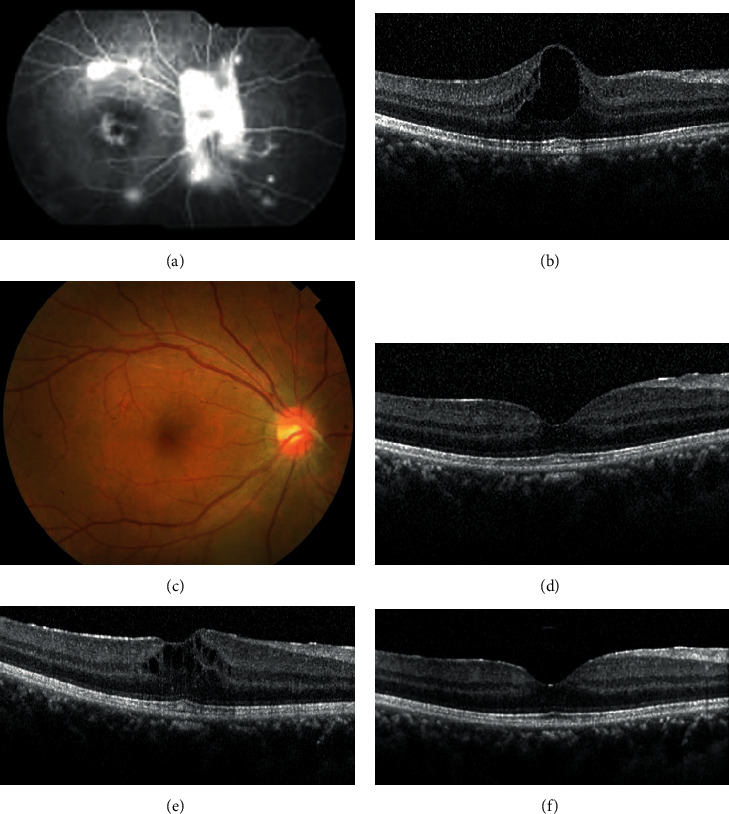
(a) FA showed vascular tortuosity, peripheral retinal NV, and ME. (b) Persistent ME after periocular depot triamcinolone acetonide injection (central foveal thickness 538 *μ*m). (c) After the first intravitreal aflibercept injection, the retinal NV regressed. (d) ME also subsided (central foveal thickness 309 *μ*m). (e) ME recurred after 3 months (central foveal thickness 438 *μ*m). (f) Intravitreal aflibercept injections had to be performed every three months to keep macula free from edema (central foveal thickness 295 *μ*m).

Case 2 was a 27-year-old female with presenting BCVA 20/30 OD due to VH. Oral transamin and topical steroid treatment were given. FA showed vascular tortuosity, peripheral retinal NV, and cystoid macular edema (CME) (Figure 2(a)). Intravitreal aflibercept was used as rescue therapy due to persistent CME and retinal NV after administration of periocular depot triamcinolone acetonide injection and topical nepafenac (Figure 2(b)). After the first intravitreal aflibercept injection, the retinal NV regressed (Figure 2(c)) and CME subsided (Figure 2(d)). The patient's BCVA improved to 20/25. However, ME recurred after 3 months (Figure 2(e)). Thereafter, intravitreal aflibercept injections were administered once every three months to keep her BCVA 20/20 (Figure 2(f)). The patient has been followed up for 28 months. Her intraocular pressure was within normal limits during the whole course. The treatment was well tolerated by both patients without significant adverse events.

## 3. Discussion

Eales disease was first described in 1880, and idiopathic inflammatory disorder of the retinal vessels (primarily veins) was presumed thereafter [1–3]. Recurrent vitreous and retinal hemorrhages resulted in sudden visual impairment. It is thought to be related to tuberculosis and is predisposed in certain geographic areas and age groups (15 to 40 years) [6].

Currently, the most favored etiologies of Eales disease are tuberculosis and hypersensitivity to tuberculoprotein [6]. 50-70% of epiretinal membrane samples were found positive for *Mycobacterium* species by polymerase chain reaction (PCR) analysis. MPB64 gene of *M. tuberculosis* has been found in a significant number of well-documented Eales disease patients. However, cultures of vitreous specimen did not show growth of *M. tuberculosis* in any of the vitreous aspirates. Thus, although Eales disease patients may not carry viable organisms, they may harbor nonviable organisms or deoxyribonucleic acid (DNA) of *M. tuberculosis* [7].

No recent studies have been able to definitively differentiate Eales disease from tuberculous vasculitis, though some association exists between this type of occlusive vasculitis and tuberculosis. Negative results of QuantiFERON®-TB tests ruled out ocular tuberculosis in the two patients reported here. Moreover, a negative workup, including the use of MRI to differentiate optic neuritis-induced retinal NV, in such cases, could indicate idiopathic occlusive vasculitis [8–10].

The management of Eales disease includes medical therapy, intravitreal injections, photocoagulation, and surgery [7, 11–13]. Corticosteroids are the mainstay of therapies used in the active perivasculitis stage to control inflammation. In patients with hypersensitivity to tuberculoproteins (positive Mantoux test), antitubercular treatment should also been given for 9 months. Panretinal photocoagulation can be performed for ischemic retina.

In cases of macular involvement, periocular depot triamcinolone acetonide injections can be attempted first. If ME or VH persists, treatment with intravitreal anti-VEGF agent such as bevacizumab (Avastin, Genentech Inc., South San Francisco, CA) has been reported [11, 12]. Massive VEGF expression observed in Eales disease might be due to retinal ischemia and chronic low-grade inflammation [4, 5]. Intravitreal bevacizumab has been effective in regressing NV, hastening the resolution of VH, or reducing the need for vitrectomy. Intravitreal bevacizumab as an adjunct to vitrectomy in advanced Eales disease has also been advocated [10]. However, it might increase the risk of tractional retinal detachment.

To the best of our knowledge, aflibercept has not been previously used in the treatment of Eales disease. In case 1, intravitreal ranibizumab was first administered but showed no obvious response. Therefore, aflibercept was used and showed effects in regressing retinal NV. In case 2, only aflibercept was used and showed favorable results. Our explanation for the better response of intravitreal aflibercept treatment was that aflibercept binds not only all isoforms of VEGF-A, and VEGF-B, but also placental growth factor (PGF). In addition, aflibercept had faster association rate and higher binding affinity for VEGF than ranibizumab. Aflibercept thus may neutralize intravitreal VEGF level more effectively and had further positive effect on reduced inflammation [ 14, 15].

Intravitreal aflibercept appeared effective in regressing retinal NV in Eales disease. Because there may be bleeding or ME on more than one occasion in these patients, repeated injections were necessary in cases of recurrence. This study demonstrated the rescue effects of intravitreal aflibercept in the treatment of persistent retinal NV and ME in Eales disease. More cases and long-term follow-up are mandatory to further elucidate the mechanisms involved and quantitate the therapeutic potential.

## Data Availability

The data used to support the findings of this study are available from the corresponding author upon request.
